# Effects of Intrinsic Properties on Fracture Nucleation and Propagation in Swelling Hydrogels

**DOI:** 10.3390/polym11050926

**Published:** 2019-05-27

**Authors:** Jingqian Ding, Ernst W. Remij, Joris J. C. Remmers, Jacques M. Huyghe

**Affiliations:** 1 Department of Mechanical Engineering, Eindhoven University of Technology, P.O. BOX 513, 5600 MB Eindhoven, The Netherlands; jingqian.ding@outlook.com (J.D.); ernst_remij@hotmail.com (E.W.R.); j.j.c.remmers@tue.nl (J.J.C.R.); 2 Bernal Institute, University of Limerick, Limerick, V94 T9PX, Ireland

**Keywords:** hydrogel, swelling, fracture, nucleation, propagation

## Abstract

In numerous industrial applications, the microstructure of materials is critical for performance. However, finite element models tend to average out the microstructure. Hence, finite element simulations are often unsuitable for optimisation of the microstructure. The present paper presents a modelling technique that addresses this limitation for superabsorbent polymers with a partially cross-linked surface layer. These are widely used in the industry for a variety of functions. Different designs of the cross-linked layer have different material properties, influencing the performance of the hydrogel. In this work, the effects of intrinsic properties on the fracture nucleation and propagation in cross-linked hydrogels are studied. The numerical implementation for crack propagation and nucleation is based on the framework of the extended finite element method and the enhanced local pressure model to capture the pressure difference and fluid flow between the crack and the hydrogel, and coupled with the cohesive method to achieve crack propagation without re-meshing. Two groups of numerical examples are given: (1) effects on crack propagation, and (2) effects on crack nucleation. Within each example, we studied the effects of the stiffness (shear modulus) and ultimate strength of the material separately. Simulations demonstrate that the crack behaviour is influenced by the intrinsic properties of the hydrogel, which gives numerical support for the structural design of the cross-linked hydrogel.

## 1. Introduction

As industrial applications require computational tools that resolve the microstructure of products rather than tools that smooth out microstructures, increasingly higher levels of versatility and robustness are demanded of finite element codes. Indeed, heterogeneities, discontinuities, microcracking, and contact problems can potentially complicate a microstructural computation. As an example of a such a computation, in this paper we study the swelling of hydrogel beads. Swelling or drying volume transitions of ionized hydrogels can be induced by a continuous change of various conditions, such as temperature, pH, electric field, and salt concentration [1]. The degree of volume transformation depends on the composition and structure of the cross-linked network [2,3]. Because of their swelling behaviour, ionized hydrogels have received considerable attention for pharmaceutical and industrial applications, such as drug delivery or disposable diapers. Many swelling processes of hydrogels start from a dry state. When the dry hydrogel is placed in a solvent, it absorbs fluid and swells. During this volume transformation, a stress field is created within the hydrogel, and to some degree, cracks are generated and developed [4,5].

As the US patent US7517586B2 addressed, hydrogel-forming polymers used as absorbents require adequately high sorption capacity and gel strength [6]. Gel strength resists deformation of hydrogel particles during swelling and avoids gel blocking within a swollen gel bed, which is achieved by increasing the level of surface cross-linking. Although a surface cross-linked hydrogel increases gel stiffness at the surface, it reduces the absorbent capacity and increases the tendency to have a brittle fracture. It is crucial to optimize the balance between absorbent capacity and gel strength.

The effect of the microstructure of the material on swelling capacity has been well-tested; however, few studies take the effects of damage into consideration. Cervera et al. [7] developed a computational model to analyse the progressive cracking due to the swelling of concrete in large concrete dams. Zhang et al. [8] numerically studied the phenomenon of crack healing induced by swelling in gels. Guo et al. [9] found out that the intrinsic properties of the interface between a polymeric hydrogel and a rigid substrate have a great influence on the opening profile of the interface crack. Although the studies mentioned above deal with crack behaviours induced by swelling, there is still a long way to go. Considering that the structural optimization of the hydrogel is largely affected by the intrinsic properties of the cross-linked and original hydrogel, it is important to study the effects of material properties on crack behaviours.

The extended finite element method (XFEM) is an efficient tool used to simulate fracture growth without re-meshing. It was first applied by Belytschko and Black [10] by adding an additional degree of freedom on nodes which belong to the element crossed by the discontinuity. Wells and Sluys [11] incorporated a cohesive surface formulation into the method to achieve the crack propagation in any arbitrary direction. Similarly, Leonhart and Meschke took the moisture transport in opening discontinuities into account and analysed the crack propagation in partially saturated porous media [12]. Kraaijeveld et al. [13] took osmotic forces into consideration and studied Mode I crack propagation in saturated ionized porous media in small deformations. Irzal et al. [14] extended the partition of unity approach of fracturing porous media into the finite deformation regime. In order to capture the pressure gradient across the discontinuity, Remij et al. [15] developed the enhanced local pressure model (ELP): a separate degree of freedom for the pressure in the discontinuity was added to the pressure left and right of the crack. Furthermore, the level set method (LSM) [16] is commonly incorporated with XFEM to model crack growth. LSM is used to locate the crack and its tip, and it simplifies the selection of the enhanced nodes in XFEM.

In the present work, a finite deformation model is presented to study the crack behaviour of a heterogeneous swelling hydrogel. We integrated XFEM and ELP to capture the pressure difference and fluid flow between the crack and the hydrogel, and used the cohesive zone method to achieve crack propagation without re-meshing [17].

## 2. Results

### 2.1. Crack Propagation

**Figure 1:**
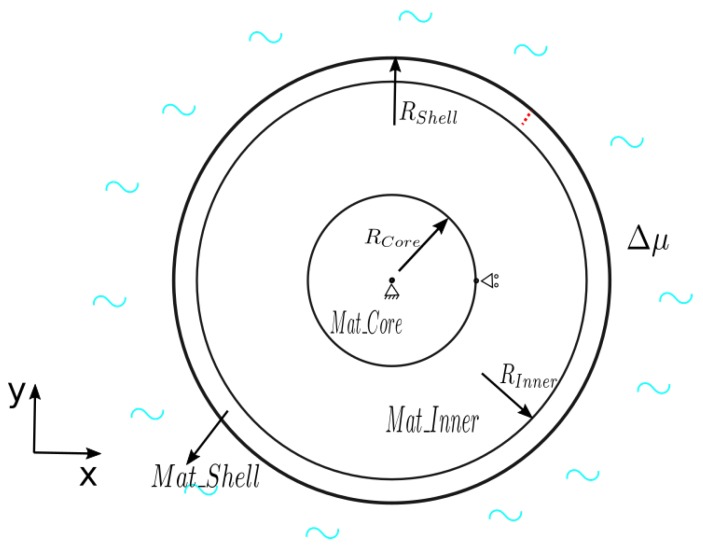
The geometry and boundary conditions of the sample. A changing chemical potential is applied along the outer surface. The red dashed line indicates the initial crack. ($R_{Shell}=0.5$ mm, $R_{Inner}=0.45$ mm, $R_{Core}=0.2$ mm).

Here, we consider a circular sample consisting of three different materials (Figure 1). We fixed the middle point and constrained the node located on the boundary between the core and middle part at $0^{{}^{\circ}}$ in the y direction. A changing chemical potential was applied at the outer surface, which led to the swelling of the medium. The material properties are listed in Table 1.

**Figure 2:**
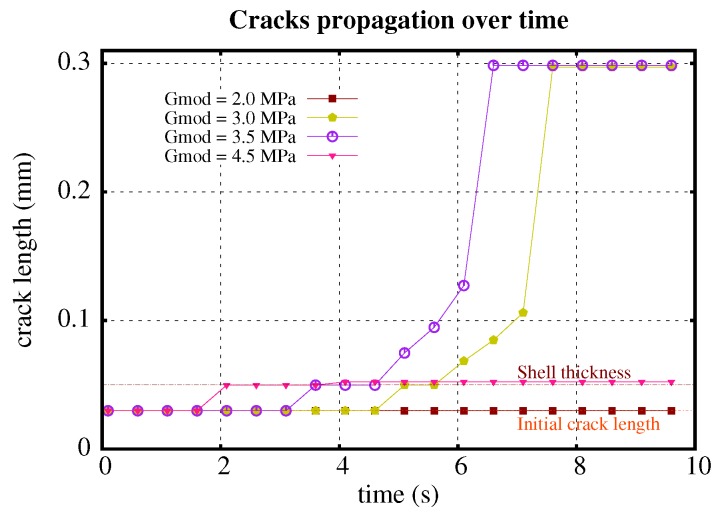
Crack propagation profile over time with different stiffnesses of the shell.

**Figure 3:**
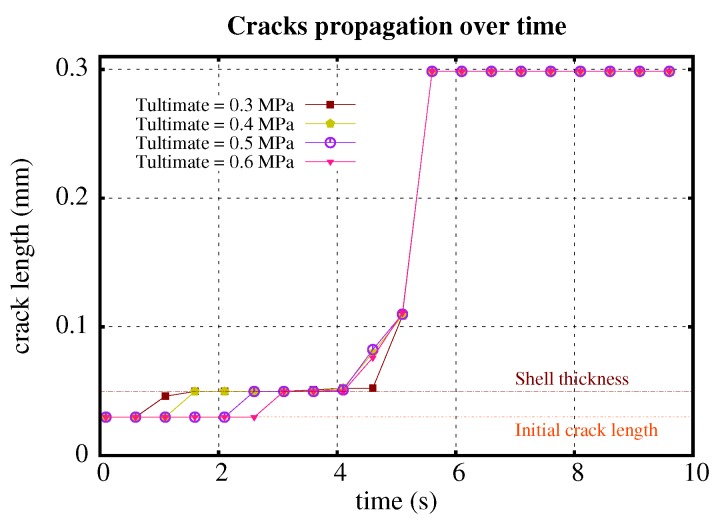
Crack propagation profile over time with different ultimate strengths of the shell.

**Figure 4:**
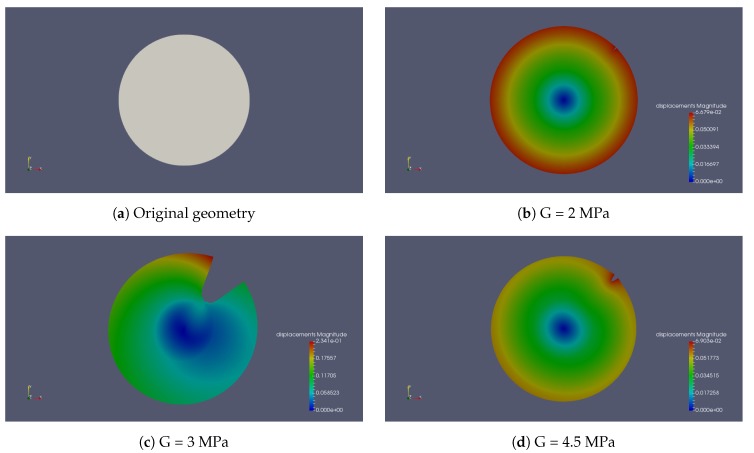
The displacement profile with different shear moduli of the shell.

Figure 2 and Figure 3 show the behaviour of crack propagation with different stiffnesses and ultimate strength of the shell. The crack propagates over time for four different shell shear moduli (2 MPa, 3 MPa, 3.5 MPa and 4.5 MPa) and the same shell ultimate strength of 0.52 MPa (Figure 2). For G=2.0 MPa, the initial crack opens without propagation. For the other four examples, every initial crack propagates. Generally, a stiffer material reaches high stress faster than a softer material with the same amount of deformation, causing material failure and crack growth. The higher the shear modulus is, the earlier the initial crack propagates, as seen in Figure 2 with crack propagation plotting for G=3.0,3.5, and 4.5 MPa. At the same time, a stiffer shell resists the particle’s deformation and helps to keep the shape of the particle. Less deformation comes with smaller stress in the middle part, which suppresses the crack growth (Figure 4). This is the reason why the crack length of G=4.5 MPa is much smaller than with G=3.0 MPa and G=3.5 MPa.

Similarly, we fixed the shell shear modulus ($G_{shell}=$ 4 MPa), and studied the effect of various ultimate strengths (0.3 MPa, 0.4 MPa, 0.5 MPa, and 0.6 MPa) of the middle material (Figure 3). The effect of ultimate strength is straightforward; the ultimate strength does not contribute to the propagating length of the crack, but it increases the capacity of the material to resist tension, and only affects the rate of propagation. The higher the ultimate strength, the later the crack starts to propagate. It is advisable to achieve relatively high ultimate strength in the material design to obtain higher elongating resistance.

### 2.2. Crack Nucleation

The energy accumulates within the hydrogel particle when it swells. There are two ways to dissipate energy, propagate existing cracks, or nucleate new cracks. In Section 2.1 we discussed the behaviour of crack propagation. In the current section, we discuss the behaviour of crack nucleation.

The same geometry (Figure 1) and material properties (Table 1) are used here to model crack nucleation. In order to avoid too many cracks nucleating at the same time too close together, we made an extra constraint that there were at least 30 elements between two cracks. We compared the nucleation state within three groups: different shear moduli of the shell, different ultimate strengths of the shell, and different shear moduli of the middle part of the sample.

**Figure 5:**
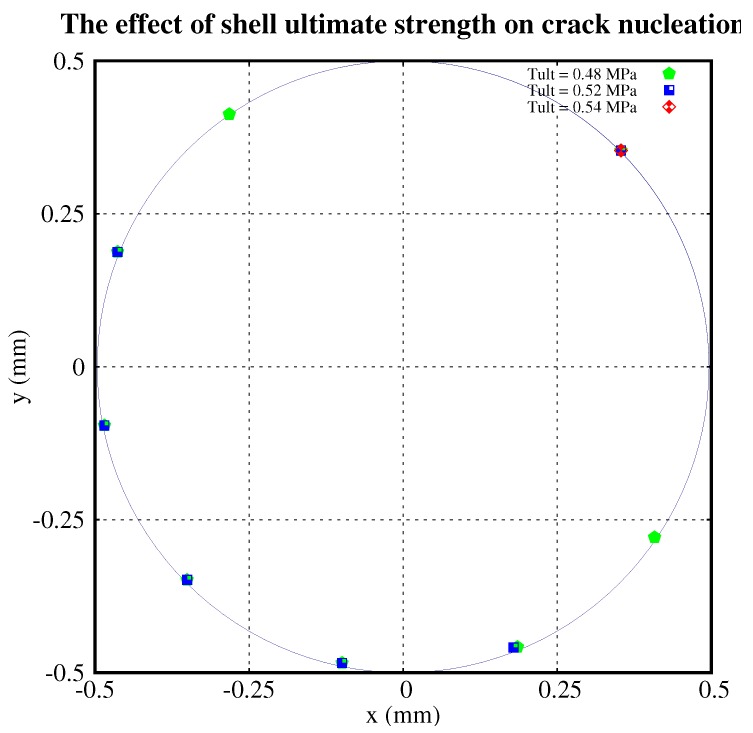
Crack nucleations with different ultimate strengths of the shell. Every coloured dot represents one nucleation site. The results of three separate computations with different values of the shell’s ultimate strength are superimposed. The colour of the dot specifies the computation to which it belongs.

The point at $45^{{}^{\circ}}$ to the x-axis in the first quadrant is the initial crack. In Figure 5, we plotted the nucleations with the shell’s ultimate strength of 0.48 MPa, 0.52 MPa, and 0.54 MPa, respectively. There was no nucleation with an ultimate strength of 0.54 MPa. For $\tau_{ult}=0.52$ MPa, it has five nucleations. When $\tau_{ult}$ decreases to 0.48 MPa, the nucleations increase to 7.

**Figure 6:**
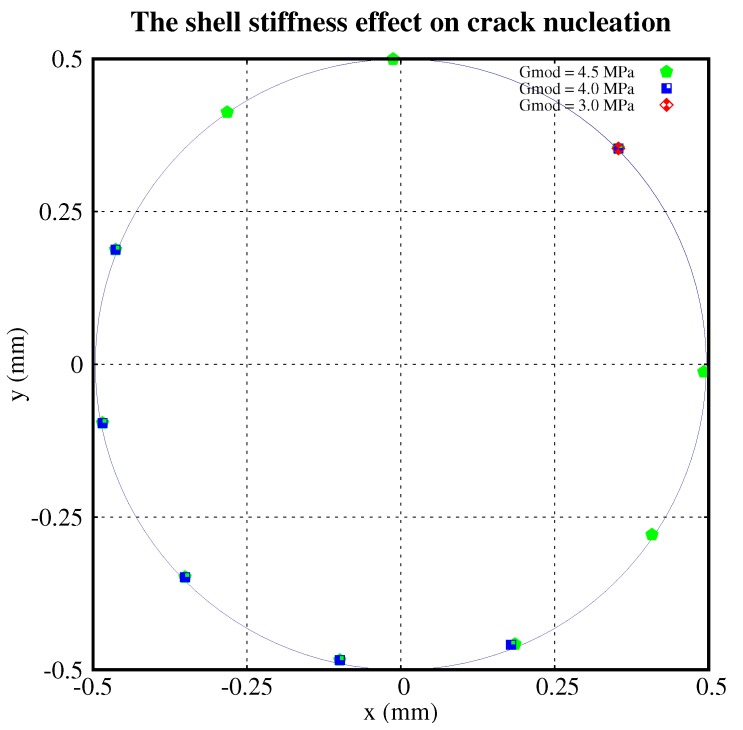
Crack nucleations with different stiffnesses of the shell.

**Figure 7:**
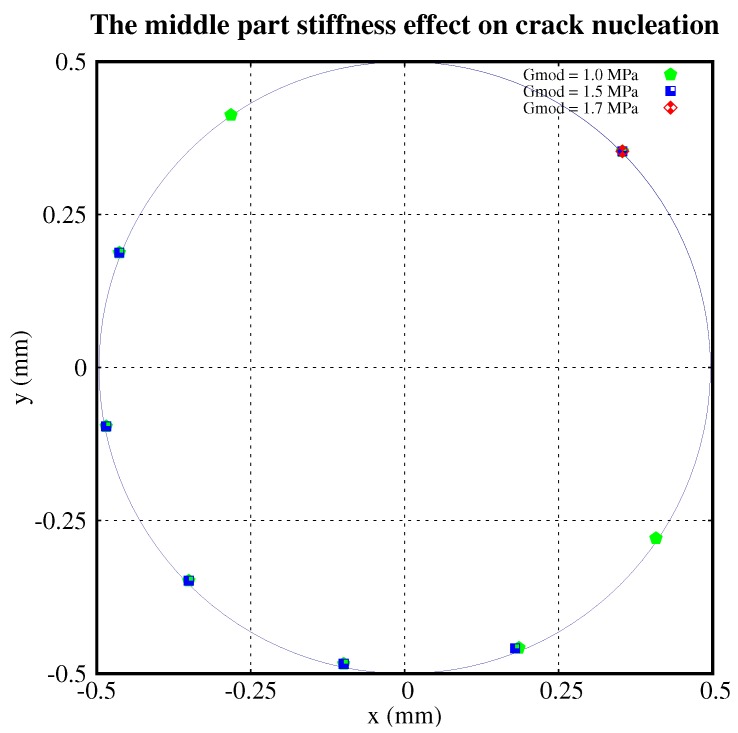
Crack nucleations with different stiffnesses of the middle part.

Figure 6 shows the nucleation locations with different shear moduli of the shell. There were nine nucleations for G=4.5 MPa and five nucleations for G=4.0 MPa. No new cracks nucleated for *G* = 3.0 MPa. Similarly, Figure 7 plots the nucleation locations with different shear moduli of the middle part of the particle. It shows that there are 7, 5, and 0 nucleations relating to G=1.0,1.5and1.7 MPa, respectively. The distribution of nucleations is roughly symmetric about the diameter crossing the initial crack.

**Figure 8:**
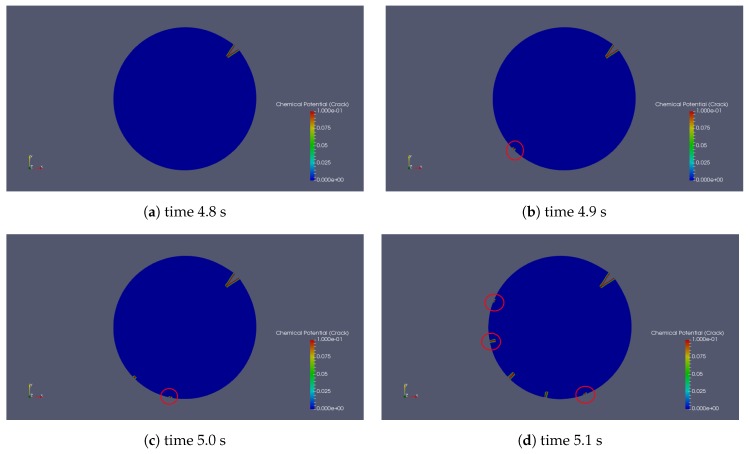
Nucleations at different times (s), red circles emphasize nucleation spots.

Figure 8 is the chemical potential distribution within the crack with different time steps. The nucleation process shows a cascade phenomenon (Figure 8). At the time of 4.8 s, the initial crack propagates without any nucleation. After 0.1 s, there is one nucleation. Another new crack nucleated after 0.1 s. At the time of 5.1 s, the previous two nucleations kept growing, and another three new cracks were generated.

## 3. Discussion

This study illustrates how the relationship between the microstructure and function of a product can be studied using a dedicated nonlinear multiphysics, multicomponent, finite element analysis. The type of analysis done in this study was made possible with commercial codes, because they lack the vital options needed. The feasibility of complex non-linear analyses combining large deformations and mesh-free multiple crack propagations through a swelling heterogeneous material has been demonstrated. Much more is needed for the robustness of the XFEM code in computations that resolve the microstructure, as deformations are very large, locally, and heterogeneities hamper the smoothness of the solutions. This investigation is mainly focused on the effects of intrinsic properties on the fracture behaviour. We studied two dissipative mechanisms here, where one is the propagation of existing cracks, and the other is the nucleation of cracks. From the results, it appears that the crack propagation and nucleation are largely affected by the intrinsic properties of the material, and particularly the properties of the cross-linked shell around the softer swelling hydrogel particle.

Generally, the higher the shear modulus of the shell, the earlier the initial crack propagates. Besides, a stiffer shell resists the particle’s ability to deform, and helps to keep the shape of the particle. Less deformation comes with smaller effective stress in the middle part, which suppresses crack growth. However, a swelling media requires high swelling capacity. Therefore, there needs to be a balance between the swelling of the inner part and the elastic stiffness of the outer part. This numerical simulation can be used as a tool to optimize the material property of the gel in the microstructural design of the swelling hydrogel particle. By comparing the crack propagation with different ultimate strengths of the shell, we found that the ultimate strength only affects the rate of the propagation. The higher the ultimate strength is, the slower the crack propagates.

From Figure 5, Figure 6 and Figure 7, we conclude that:

- The higher the ultimate strength of the shell, the fewer the cracks which nucleate.
- The higher the shear modulus of the shell, the more cracks which nucleate.
- The higher the shear modulus of the middle part of the particle, the fewer the cracks which nucleate.
- The distribution of nucleations is roughly symmetric about the diameter crossing the initial crack.

Figure 8 presents a cascade of nucleations with different crack openings with material properties of Table 1. The events illustrate that the failure of the gel builds up in stages. It starts from fewer defects and weakens the material while the material is still functioning. When more and more defects appear and interact with each other, the material finally fails. The process of the defects is the same as the process of the nucleation. The opening of cracks not only depends on the stress state, but also on the neighbouring cracks. If the neighbouring crack is located close to the current crack with a relatively large opening, it will impede the opening of the current crack.

The software presented in this paper serves as a great numerical support for the design of a proper cross-linked shell, such as the special cross-link density required to achieve a specific stiffness and fracture resistance. The stiffness ratio between the inner part and the shell is a critical parameter that determines the performance of the product. A high ratio delays the swelling of the hydrogel, and too high a ratio disallows the failure of the hydrogel altogether.

## 4. Methods

### 4.1. Kinematic Relations

**Figure 9:**
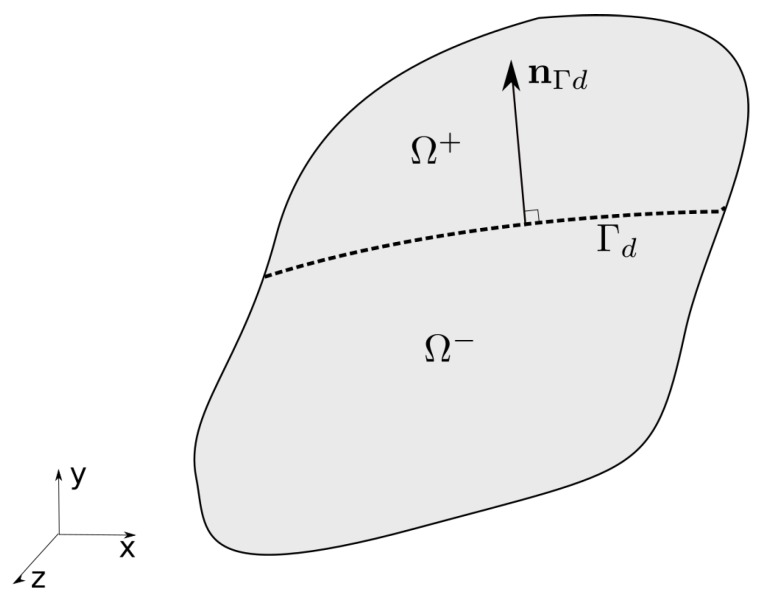
The body Ω is crossed by a discontinuity (dashed line). $n_{\Gamma_{d}}$ represents the normal of the discontinuity surface pointing to $\Omega^{+}$.

We considered a body Ω crossed by a discontinuity (Figure 9). The body was divided into two subdomains, $\Omega^{+}$ and $\Omega^{-}$. The total displacement field of the solid skeleton was described by a regular displacement field $\hat{u}$ and an enhanced displacement field $\tilde{u}$,

(1) $$u\left(X,t\right)=\hat{u}\left(X,t\right)+H_{\Gamma_{d}}\left(X\right)\tilde{u}\left(X,t\right),$$

where X is the material point in the reference configuration of the solid, $H_{\Gamma_{d}}$ is the Heaviside step function, defined as

(2) $$H_{\Gamma_{d}}=\left\{\begin{array}{cc} 1 & \;X\in \Omega^{+}\\ 0 & \;X\in \Omega^{-} \end{array}\right..$$

The chemical potential field is discontinuous across the discontinuity and the hydrogel, and defined as

(3) $$\mu^{f}\left(X,t\right)={\hat{\mu }}^{f}\left(X,t\right)+H_{\Gamma_{d}}\left(X\right){\tilde{\mu }}^{f}\left(X,t\right),$$

In the discontinuity, the chemical potential is equal to an independent variable $\mu_{d}$,

(4) $$\mu^{f}=\mu_{d},\;X\in \Gamma_{d}.$$

Hence, the value of the chemical potential jumps from ${\hat{\mu }}^{f}$ to $\mu_{d}$ to ${\hat{\mu }}^{f}+{\tilde{\mu }}^{f}$ as one crosses the discontinuity from $\Omega^{-}$ to $\Omega^{+}$.

### 4.2. Balance Equations

We considered the body as a solid skeleton with fully saturated interstitial fluid. It was assumed that there was no mass transfer, and thermal gradients, inertia, and gravity were neglected. Based on Biot’s theory, the momentum balance reads

(5) ∇·σ=0inΩ,

with σ the total stress, which is decomposed into the effective stress $\sigma_{e}$ and the pore fluid pressure *p*,

(6) $$\sigma =\sigma_{e}-pI,$$

with I being the unit tensor.

Equations (5) and (6) can be written with respect to the reference configuration, using the transformation of $P=J\sigma \cdot F^{-T}$, read

$$\begin{array}{c} \nabla_{0}\cdot P=0\;\mathrm{in}\;\Omega_{0}\;(\mathrm{momentum}\;\mathrm{balance}),\\ P=P_{e}-JpF^{-T}\;(\mathrm{total}\;\mathrm{first}\;\mathrm{Piola-Kirchhoff}\;\mathrm{stress}), \end{array}$$

where $P_{e}$ is the effective first Piola-Kirchhoff stress.

Conservation of mass for an incompressible fluid yields the mass balance in the reference configuration,

(7) $$\dot{J}+\nabla_{0}\cdot Q=0,$$

with $\dot{J}=J\mathrm{div}\dot{u}$ and $Q=-K\cdot \nabla_{0}\mu^{f}$ the seepage flux obeying the Darcy’s relation in the presence of the concentration gradient. In the equation of the seepage flux, K is the permeability tensor back-transformed to the reference configuration, and $\mu^{f}$ is the chemical potential, defined as

(8) $$\mu^{f}=p-\pi ,$$

with *p* being the hydrostatic pressure, and π the osmotic potential.

The fracture process behaviour is governed by a traction separation law. Here, we assumed stress continuity from the gel to the discontinuity, the local momentum balance being described as

(9) $$P\cdot n_{\Gamma_{d}}=J\left|\left|F^{-T}\cdot n_{\Gamma_{d}}\right|\right|t_{d}-J\left(\mu_{d}^{f}+\pi_{d}\right)F^{-T}\cdot n_{\Gamma_{d}},$$

in which $n_{\Gamma_{d}}$ is the normal of the discontinuity $\Gamma_{d}$, $t_{d}$ is the traction, and $\mu_{d}^{f}$ and $\pi_{d}$ are the chemical potential and osmotic pressure, respectively, within the discontinuity.

The local mass balance was obtained by integrating the continuous mass balance across the discontinuity.

(10) $$n_{\Gamma_{d}}\cdot \left(Q_{\Gamma_{d}}^{+}-Q_{\Gamma_{d}}^{-}\right)=Ju_{n}\frac{\partial }{\partial s}\left(k_{d}\frac{\partial \mu_{d}^{f}}{\partial s}\right)-Ju_{n}\langle \frac{\partial v}{\partial s}\rangle -J{\dot{u}}_{n},$$

where $k_{d}$ is the conductivity in the discontinuity

(11) $$k_{d}=\frac{{\left(u_{n}\right)}^{3}}{12\mu },$$

with μ being the viscosity of the fluid, $u_{n}=\tilde{u}\cdot n_{\Gamma_{d}}$ the opening of the discontinuity.

### 4.3. Swelling Behaviours

Swelling equilibria between the hydrogel and the immersed fluid must fulfil $\mu_{in}=\mu_{ex}$ ($\mu_{in}$ is the chemical potential within the hydrogel, and $\mu_{ex}$ describes the chemical potential of the surrounding fluid).

The difference of the osmotic pressure between the hydrogel and the surrounding fluid allows ions or fluid to go into or out of the hydrogel, leading to swelling or shrinking. Because the ionic diffusion coefficient of SAP is two orders of magnitude larger than the pressure diffusion coefficient of SAP, we assumed the ionic constituent to be drained. Based on Van’s Hoff empirical relation, the osmotic pressure difference was given by

(12) $$\Delta \pi \left(\varepsilon \right)=RT\sqrt{{\left(c^{fc}\right)}^{2}+4{\left(c^{ex}\right)}^{2}}-2RTc^{ex},$$

where *R* is the gas constant, *T* is the temperature, $c^{fc}$ is the fixed charge density, and $c^{ex}$ is the external salt concentration.

### 4.4. Nucleation Mechanism

Crack nucleation and growth are part of the fracture development. Nucleated micro-cracks are based on the stress state of the solid skeleton. The stress state was obtained by averaging effective stresses around the crack tip. Remmers et al. [18] used a Gaussian weighted function to calculate the averaged stress:

$$\begin{array}{cc} \sigma_{av} & =\sum_{i=1}^{n_{int}}\frac{\omega_{i}}{\omega_{tot}}\sigma_{i}\\ \omega_{tot} & =\sum_{j=1}^{n_{int}}\omega_{j}, \end{array}$$

here, $n_{int}$ is the number of integration points in the domain, and $\omega_{i}$ is the weight factor relating to the integration point *i*. The weight factor is defined as

(13) $$\omega_{i}=\frac{1}{l_{a}^{3}}e^{\frac{-r_{i}^{2}}{2l_{a}^{2}}},$$

where $l_{a}$ is the length scale parameter, and $r_{i}$ denotes the distance between the integration point *i* and the crack tip.

### 4.5. Crack Propagation Mechanism

The crack propagation is governed by the stress state of the crack tip. The stress is calculated by averaging the stress around the crack tip. The traction $t_{d}$ in Equation (9) acting on the fracture surface is based on the cohesive constitutive relation, and governs the propagation of the crack. When the averaged stress of the crack tip exceeds the ultimate strength, the crack starts to propagate. The normal traction $t_{n}$ is described as

(14) $$t_{n}=\tau_{ult}exp\left(-\frac{u_{n}\tau_{ult}}{G_{c}}\right),$$

where $\tau_{ult}$ is the ultimate strength of the material, and $G_{c}$ denotes the fracture toughness.

### 4.6. Constitutive Equation

In this paper, we used a compressible Neo-Hookean model. Though the solid itself was incompressible, due to its porous structure, the entire solid matrix was compressible. The Cauchy stress [19,20] is given by

(15) $$\sigma =-\frac{1}{6}\frac{\ln J}{J}GI\left(-1+\frac{3(J+n_{s,0})}{\left(-J+n_{s,0}\right)}+\frac{3\ln\left(J\right)Jn_{s,0}}{{\left(-J+n_{s,0}\right)}^{2}}\right)+\frac{G}{J}\left(F\cdot F^{T}-J^{2/3}I\right),$$

where *G* is the shear modulus, and $n_{s,0}$ represents the initial solid volume fraction. As *J* tends to $n_{s,0}$, the fluid content vanishes and Equation (15) becomes an incompressible law.

When osmotic swelling is included, the total stress reads

(16) $$\sigma_{tot}=\sigma -\left(\mu^{f}+\pi \right)I.$$

## Figures and Tables

**Table 1:** Material properties.

Name	Symbol	Value
Shear modulus (core)	*G*	0.05 MPa
Shear modulus (middle)	*G*	(1.0,1.5,1.7) MPa
Shear modulus (shell)	*G*	(2.0,3.0,3.5,4.0,4.5) MPa
Intrinsic permeability	$k_{int}$	1.0 × 10${}^{-11}$ mm${}^{2}$
Fluid dynamic viscosity	μ	1.0 × 10${}^{-9}$ MPas
Porosity	φ	0.83
Ultimate strength (core)	$\tau_{ult}$	0.01 MPa
Ultimate strength (middle)	$\tau_{ult}$	(0.3,0.4,0.5,0.6) MPa
Ultimate strength (shell)	$\tau_{ult}$	(0.48,0.52,0.54) MPa
Toughness	$G_{c}$	0.01 N/mm
Gas constant	R	8.3145 J·mol${}^{-1}\cdot$K${}^{-1}$
Temperature	T	293.0 K
Initial fixed charge density	$c_{0}^{fc}$	332.0 × 10${}^{-6}$ mmoleq/mm${}^{3}$
External salt concentration	$c_{ex}$	154.0 × 10${}^{-6}$ mmol/mm${}^{3}$
